# Precursor Lymphoblastic Lymphoma in the Extramedullary Tissue: A Rare Manifestation of Chronic Myeloid Leukemia in Blast Crisis

**DOI:** 10.7759/cureus.11009

**Published:** 2020-10-17

**Authors:** Huma Mansoori, Maria Faraz, Hira Qadir, Anila Rashid, Maria Ali

**Affiliations:** 1 Pathology, Dow University of Health Sciences, Karachi, PAK; 2 Health Sciences, McMaster University, Ontario, CAN; 3 Pathology and Laboratory Medicine, Aga Khan University Hospital, Karachi, PAK; 4 Transfusion Medicine, Regional Blood Centre, Karachi, PAK

**Keywords:** chronic myeloid leukemia, extramedullary, blast crisis

## Abstract

Chronic myeloid leukemia (CML) is a myeloproliferative disorder characterized by immature granulocytes in peripheral blood and bone marrow. In 95% of cases, it is always due to the presence of Philadelphia chromosome characterized by the presence of reciprocal translocation between chromosome 9 and 22. However, in 7% -17% of individuals, extramedullary proliferation also occurs, either in skin, lymph nodes, bone or central nervous system (CNS), which could be either myeloid, lymphoid or mixed progenitor in origin. The present case is of a 23-year-old male who presented with lower limb weakness, bowel and urinary incontinence. His complete blood count (CBC) findings showed a raised white blood count (WBC) of 408 X 10E9/L. Peripheral film, bone marrow biopsy and immunohistochemistry showed findings consistent with CML in chronic phase. Bone marrow cytogenetic revealed the presence of Philadelphia chromosome. Simultaneously, magnetic resonance imaging (MRI) was done which revealed extradural mass at L1-L3 level; histopathological and immunohistochemistry findings showed features compatible with precursor B cell lymphoblastic lymphoma. His cerebrospinal fluid (CSF) cytology revealed similar blast cells. This extramedullary presence of lymphoid blast cells in the CNS put the patient in the rare entity of CML in blast crisis. He was started on tablet nilotinib and also received multiple cycles of intrathecal chemotherapy with cytosar, methotrexate and hydrocortisone. He also underwent radiotherapy of extradural mass. His lower limb weakness improved dramatically. However, after receiving the fourth cycle of intrathecal therapy, the patient died consequent to neutropenic sepsis. Extramedullary blast crisis in CML has a poor prognosis. Any patient with CML, presenting with CNS symptoms or lymph node enlargement should be thoroughly investigated for extramedullary blast crisis, as there is a considerable change in management and prognosis from the prototype CML in chronic phase.

## Introduction

Extramedullary presentation of chronic myeloid leukemia (CML) in the form of myeloid sarcoma has been reported in several literature [1,2]. However concomitant presentation of CML in bone marrow along with the simultaneous presence of precursor B cell lymphoblastic lymphoma in extramedullary tissue site has rarely been cited. According to 2018, WHO classification, blast crisis of CML is defined as either more than 20% blast cells in blood/bone marrow or extramedullary proliferation of myeloid progenitor cells/ blast cells. Here, we present a rare manifestation of CML in blast crisis due to the presence of lymphoid blasts in extradural mass of central nervous system (CNS) along with granulocytic hyperplasia in peripheral blood and bone marrow.

## Case presentation

**Figure 1:**
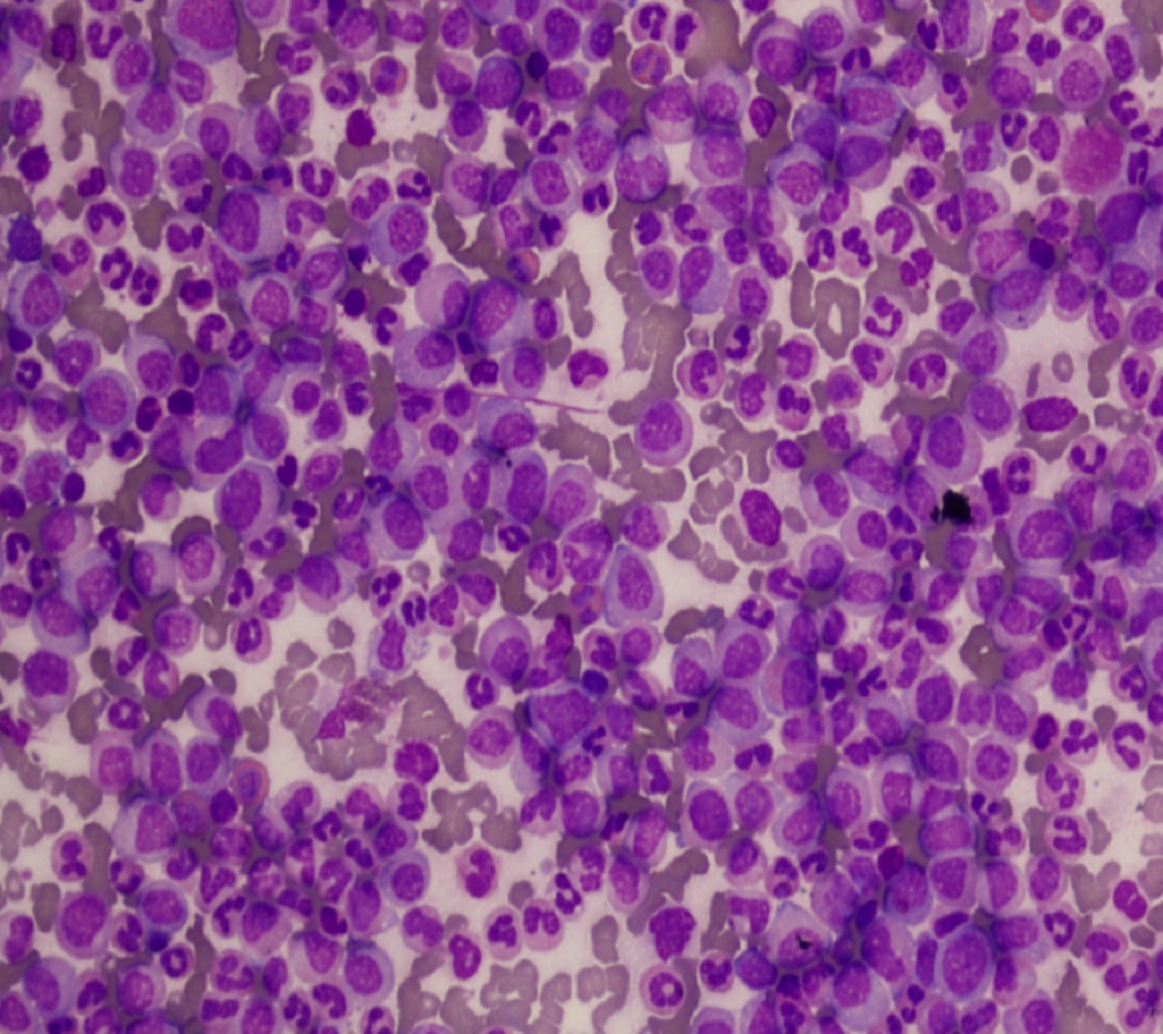
Bone marrow smear showing gross granulocytic hyperplasia

A 23-year-old male presented with bilateral limb weakness, urinary and bowel incontinence. Positive physical examination findings revealed massive splenomegaly, motor power of 1/5 and loss of deep tendon reflexes in both lower limbs. Laboratory workup revealed hemoglobin of 11.0 G/dL white cell count (WBC) of 408 x 10E9/L and platelet count of 287 x 10E9/L. Peripheral film and bone marrow findings were consistent with CML in chronic phase (Figure 1, Leishman staining; X400).

**Figure 2:**
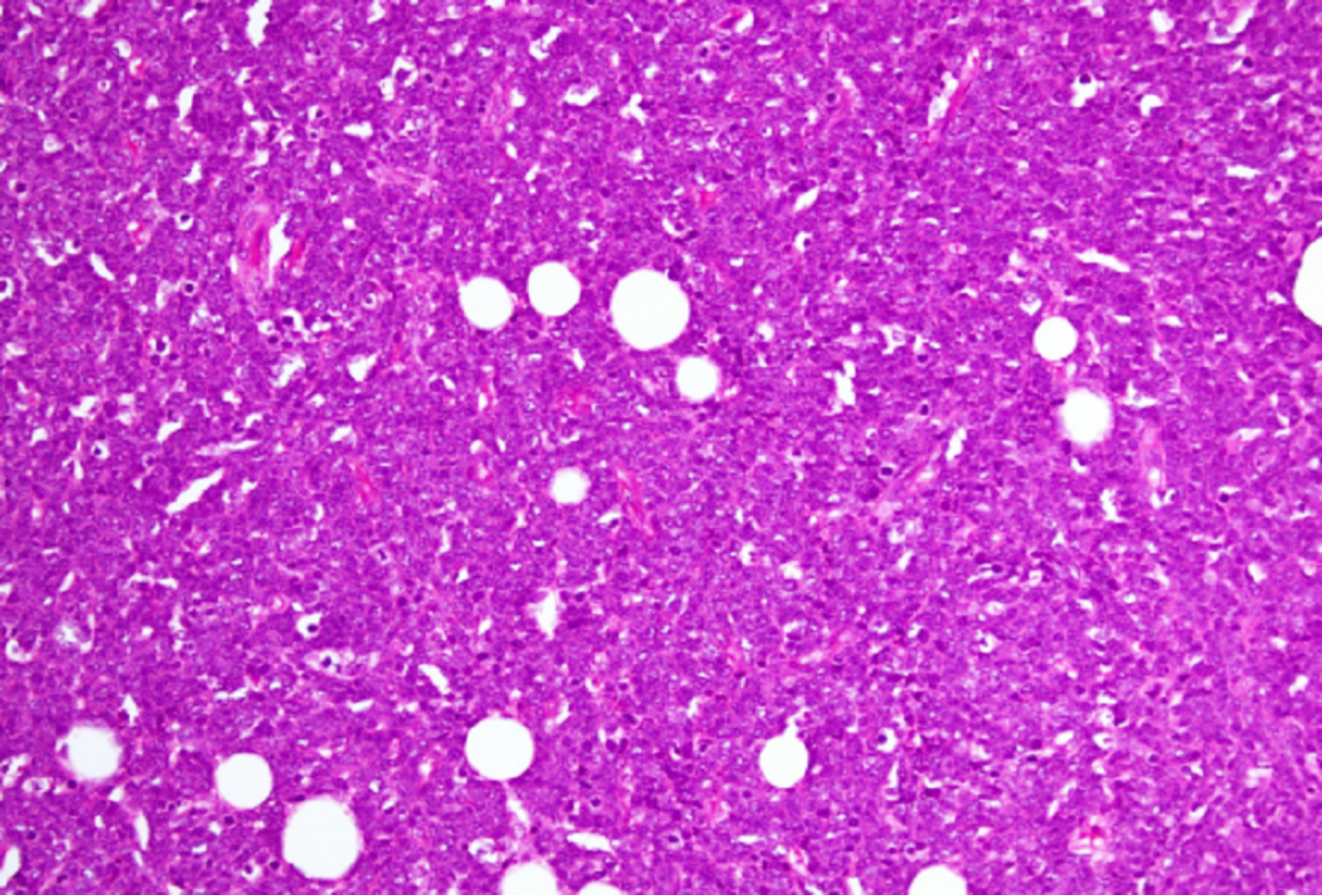
Diffuse infiltrate of lymphoid blasts in extradural mass

**Figure 3:**
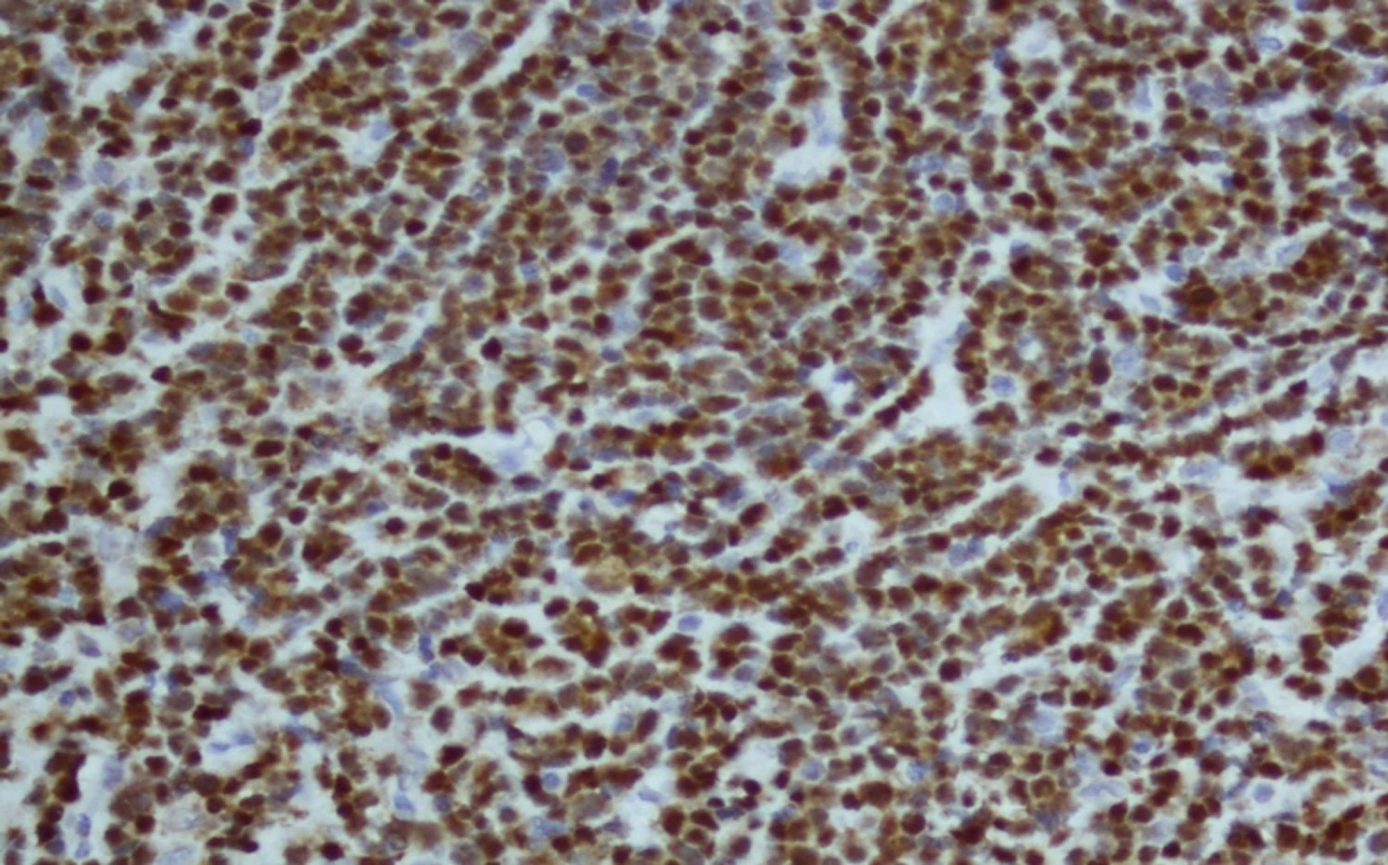
Neoplastic cells of extradural mass showing positivity for Tdt

**Figure 4:**
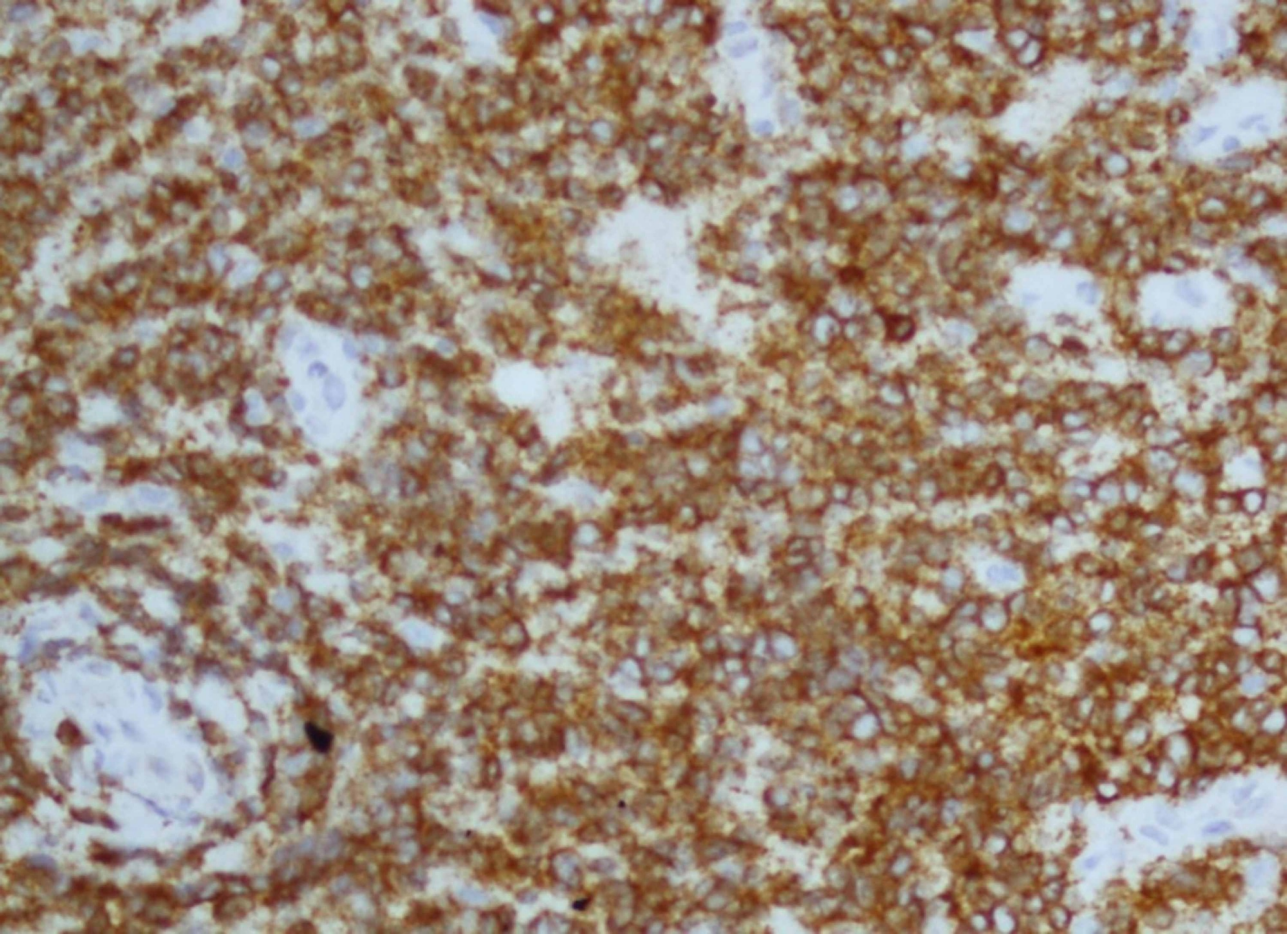
Neoplastic cells of extradural mass showing positivity for CD79a

Bcr-abl translocation was detected in 95% of nuclei counted by FISH. MRI lumbosacral spine revealed extradural mass at L1-L3 level; laminectomy was done. Histopathological findings of this extradural mass showed sheets of small-sized neoplastic cells with scant cytoplasm, round nuclei with coarse chromatin (Figure 2, hematoxylin and eosin; X200 ). Neoplastic cells were diffuse positive for Tdt and CD79a (Figures 3, 4; paraffin-embedded tissue X200), PAX5 and showed proliferative index of 95%. Cells were negative for CD3. Based on which findings were consistent with precursor B cell lymphoblastic lymphoma. 

 

His CSF DR showed WBC of 150 cu/mm, and protein of 81 mg/dL. Cytology revealed a large number of atypical cells with mildly enlarged nuclei, irregular nuclear contour and scanty cytoplasm.

This patient was diagnosed with CML with extramedullary blast crisis, presenting as CNS leukemia. Patient was started on nilotinib 800mg daily. He received multiple doses of intrathecal chemotherapy with cytosar, methotrexate and hydrocortisone followed by involved field radiotherapy of extradural mass. There was a significant improvement in lower limb weakness.

However, the patient was readmitted on third day after receiving fourth dose of triple Intrathecal therapy, with febrile neutropenia (WBC:0.2) and died after two days due to E-coli sepsis.

## Discussion

CML is the myeloproliferative disorder identified by the unopposed reproduction of the pluripotent myeloid stem cell [3]. CML has most commonly three stages: CML chronic phase, CML accelerated phase, and CML blast crisis/extramedullary phase [3]. The extramedullary phase is a relatively uncommon event with an incidence rate of 3%-7.9% [3]. It happens due to the invasion of blast cells in regions other than bone [4]. Extramedullary blast presentation can be manifested as an appearance of new tumors or enlargements of lymph nodes [5]. The manifestation of the extramedullary blast phase as a myeloid sarcoma is reported in the literature [1]. However, the occurrence of Philadelphia chromosome-positive lymphoma is quite rare [6]. Upon search of the PubMed database, we found that there is only one case reported as Philadelphia chromosome-positive B cell lymphoblastic lymphoma in testicular tissue [7]. Wei et al. found 10 case reports from 2003 to 2013 with patients having CML with the finding consistent to lymphoma in extramedullary tissue, which later found to be blast crisis only [6].

Our patient is also another case of simultaneous occurrence B cell lymphoblastic lymphoma in the paravertebral region and CML.

On review of literature, it is studied that CML is one of the risk factors for the occurrence of other cancers (solid and hematological) secondary to bcr-abl aberrations [8]. Bcr-abl gene arises due to the translocation of the gene between long arm of chromosome 9 to chromosome 22. This leads to the Philadelphia chromosome (the hallmark of CML). Gajendra et al suggested that this bcr-abl aberration has the potential to give rise to other malignancies due to genetic instability and clonal evolution [8]. Yap et al. proposed the concept of tumor heterogeneity and sub-clonal evolution and compared this tumor heterogeneity with the growing tree model [9]. The progenitor founding mutation of the tumor mainly harbors the trunk and is found in every tumor subclone. Tumor subclone is represented as the branches of the tree. They possess founding mutation and could also evolve into other somatic aberration which is known as sub-clonal evolution. which might lead to the rise of another type of tumor. This phenomenon is called sub-clonal evolution [9]. Anderson in 2011 approved this phenomenon by analyzing ALL cells through FISH and identifying the recurring genetic mutation in a different subclone of the same patient [10]. Moreover, immunophenotypic loss or gain of marker which is a part of tumor heterogeneity along with sub-clonal evolution can give rise to the metastatic subclone which is different from the primary clonal mutation [7]. The exact phenomena happened with our patients. Therefore, in CML patient Bcr-abl gene instability could rise to other tumor and it is important to have an adequate follow-up in patients for the formation of other hematological tumors secondary to sub-clonal evolution.

## Conclusions

This report suggests that Bcr-abl gene is present in all cases of CML and in 20% of acute lymphoblastic lymphoma cases may have an important role in the simultaneous presentation of dual pathology in this patient. Hence it is pivotal to keep an eye and have a regular follow-up to identify any concomitant tumor formation in the patient.

## References

[1] Campidelli C, Agostinelli C, Stitson R, Pileri SA (2009). Myeloid sarcoma: extramedullary manifestation of myeloid disorders. Am J Clin Pathol.

[2] Druker BJ, Sawyers CL, Kantarjian H (2001). Activity of a specific inhibitor of the BCR-ABL tyrosine kinase in the blast crisis of chronic myeloid leukemia and acute lymphoblastic leukemia with the Philadelphia chromosome. N Engl J Med.

[3] Yashima-Abo A, Satoh T, Abo T (2005). Distinguishing between proliferating nodal lymphoid blasts in chronic myelogenous leukemia and non-Hodgkin lymphoma: report of three cases and detection of a bcr/abl fusion signal by single-cell analysis. Pathol Int.

[4] Sahu KK, Malhotra P, Uthamalingam P (2016 Jun). Chronic myeloid leukemia with extramedullary blast crisis: two unusual sites with review of literature. Indian J Hematol Blood Transfus.

[5] Takimoto Y, Tanabe O, Kuramoto A, Sasaki N, Nanba K (1994). Hodgkin’s disease associated with chronic myeloid leukemia. Determination of bcr-abl rearrangement in paraffin-embedded tumors using the polymerase chain reaction. Acta Haematol.

[6] Zhu J, Zhang S, Zhu L (2015). Primary testicular Ph-positive B lymphoblastic lymphoma: an unusual presentation and review. Cancer Biol Ther.

[7] Wei J, Huang M, Wang Y, Zhou J (2013). Sudden extramedullary blast crisis of chronic myeloid leukemia manifesting as T-cell lymphoblastic lymphoma. Oncol Res Treat.

[8] Gajendra S, Sharma A, Sharma R, Gupta SK, Sood N, Sachdev R (2019). Hodgkin lymphoma in a case of chronic myeloid leukemia treated with tyrosine kinase inhibitors. Turk Patoloji Derg.

[9] Yap TA, Gerlinger M, Futreal PA, Pusztai L, Swanton C (2012). Intratumor heterogeneity: seeing the wood for the trees. Sci Transl Med.

[10] Anderson K, Lutz C, van Delft FW (2011). Genetic variegation of clonal architecture and propagating cells in leukaemia. Nature.

